# Ethyl pyruvate attenuates formalin-induced inflammatory nociception by inhibiting neuronal ERK phosphorylation

**DOI:** 10.1186/1744-8069-8-40

**Published:** 2012-05-28

**Authors:** Min Jung Lee, Minhee Jang, Hyuk-Sang Jung, Sung-Hoon Kim, Ik-Hyun Cho

**Affiliations:** 1 Department of Anatomy, College of Oriental Medicine, and Institute of Oriental Medicine, Kyung Hee University, Seoul, 130-701, Republic of Korea; 2 Cancer Preventive Material, Development Research Center, College of Oriental Medicine, Kyung Hee University, Seoul, 130-701, Republic of Korea

**Keywords:** Ethyl pyruvate, Inflammatory nociception, c-Fos, Phospho-ERK, PD-98059

## Abstract

**Background:**

Ethyl pyruvate (EP) possesses anti-inflammatory activity. However, the potential anti-nociceptive value of EP for the treatment of the inflammatory nociception is largely unknown. We investigated whether EP could have any anti-nociceptive effect on inflammatory pain, after systemic administration of EP (10, 50, and 100 mg/kg, i.p.), 1 hour before formalin (5%, 50 μl) injection into the plantar surface of the hind paws of rats.

**Results:**

EP significantly decreased formalin-induced nociceptive behavior during phase II, the magnitude of paw edema, and the activation of c-Fos in L4-L5 spinal dorsal horn. EP also attenuated the phosphorylation of extracellular signal-regulated kinase (ERK) in the neurons of L4-L5 spinal dorsal horn after formalin injection. Interestingly, the i.t. administration of PD98059, an ERK upstream kinase (MEK) inhibitor, completely blocked the formalin-induced inflammatory nociceptive responses.

**Conclusions:**

These results demonstrate that EP may effectively inhibit formalin-induced inflammatory nociception via the inhibition of neuronal ERK phosphorylation in the spinal dorsal horn, indicating its therapeutic potential in suppressing acute inflammatory pain.

## Introduction

Pyruvate (CH_3_COCOO^−^), the anionic form of a simple alpha-keto acid, plays a key role in intermediary metabolism as a product of glycolysis and as the starting substrate for the tricarboxylic acid (TCA) cycle [1,2]. Pyruvate is also an important endogenous scavenger of hydrogen peroxide (H_2_O_2_) and other reactive oxygen species (ROS), and an anti-inflammatory agent [1-3]. However, its poor stability in solution may limit its use as a therapeutic agent.

Ethyl pyruvate (EP), a stable and lipophilic derivative of pyruvate, has therapeutic potential in improving survival and/or ameliorating organ dysfunction in a wide variety of preclinical models of critical illnesses, such as hemorrhagic shock, severe sepsis, acute respiratory distress syndrome, acute pancreatitis, and intestinal, renal, or hepatic injuries in ischemic animal models [1-5]. Also, EP has neuroprotective effects against ischemic/traumatic brain injury [6-9], Parkinson’s disease [10,11], hypoxic-ischemic brain injury [12], and spinal cord ischemic/traumatic injury [13,14]. EP has additionally been reported to exert an anti-inflammatory effect in RAW 264.7 macrophage-like cells and lipopolysaccharide (LPS)-induced BV2 microglial cells by suppressing the activation of the nuclear factor-kappa B (NF-κB), extracellular signal-regulated kinase (ERK), and p38 mitogenactivated protein kinase (MAPK) pathways [15,16]. Recently, we reported that EP attenuates kainic acid-induced hippocampal neuronal death through its anti-inflammatory effects [17], and that the anti-inflammatory actions of EP include inhibiting ROS-dependent STAT signaling in activated microglia [18]. These findings raise the possibility that EP may behave as a potential effecter in other disease models.

Phosphorylation of ERK, a MAPK subfamily members, occurs in spinal dorsal horn (DH) neurons in response to injury and inflammation induced hyperalgesia of the peripheral tissue [19-21], and in a murine model of visceral pain [22,23]. Interestingly, phospho (p)-ERK is induced in spinal DH neurons immediately after nerve injury (10 min to 6 h), in microglia cells 2 days after injury, and in astrocytes 3 weeks later [24]. This sequential induction of p-ERK in different cell types at different times is important for neuropathic pain development at different phases [24]. Intrathecal (i.t.) injection of specific inhibitor, which specifically attenuates ERK activity, reduces nociceptive response behavior in inflammatory pain and CFA-induced joint inflammation [25], and reduces visceral pain caused by intracolonic capsacin [26]. These studies suggest an essential role of ERK in the development and maintenance of inflammatory or neuropathic hyperalgesia [19,20,27]. However, very little is known about the possible link, molecular signaling mechanisms, between p-ERK and EP evoked by an acute inflammatory pain.

The present study addressed the role of EP on spinal ERK in modulating acute inflammatory pain. The study hypothesis was that EP attenuates formalin-induced inflammatory nociception by inhibiting the phosphorylation of the neuronal ERK in the spinal cord.

## Results

### EP inhibits phase II, but not phase I, formalin-induced nociceptive response

**Figure 1:**
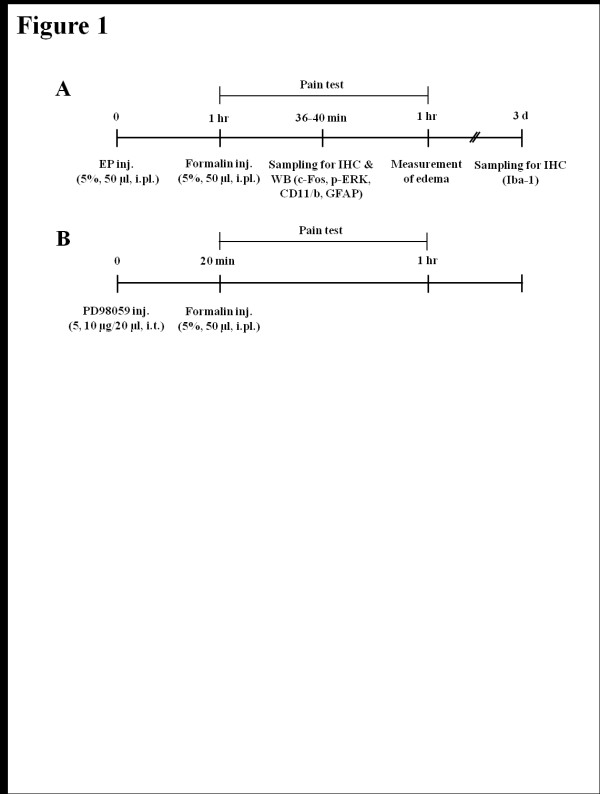
**Change of nociceptive response and hind paw edema following ethyl pyruvate (EP) administration.(A)** Effects of the systemic administration of ethyl pyruvate (EP) (10, 50, and 100 mg/kg) on paw licking and lifting responses following intraplantar injection of formalin (5%, 50 μl) into the hind paw. The rats receiving saline vehicle showed typical biphasic nociceptive behavior. While phase I nociceptive response was similar between the saline- and EP-injected rats, phase II nociceptive behavior was significantly reduced by EP administration 1 hour prior to formalin injection. All results are presented as mean ± SEM. Student's *t* test was performed at each time point after formalin injection. Values are expressed as mean ± SEM. *P < 0.01; **P < 0.05 vs. control rats (saline-pretreated and formalin-treated). Saline (n = 15), EP 10 mg/kg (n = 14), 50 mg/kg (n = 10), and 100 mg/kg (n = 13). **(B)** Total time of nociceptive behaviors during phase II. Total times of licking and lifting were attenuated by EP in a dose-related fashion following intraplantar injection of formalin. Values are expressed as mean ± SEM. *P < 0.01 vs. control rats (saline-pretreated and formalin-treated). **(C)** Effects of EP on the magnitude of hind paw edema following formalin injection. An index of paw edema was calculated as the mean difference of paw thickness (thickness of the ipsilateral paw after injection/thickness of the ipsilateral paw before injection × 100). EP significantly reduced formalin-induced edema compared to the control. Values are expressed as mean ± SEM. *P < 0.01 vs. control rats (saline-pretreated and formalin-treated).

Plantar injection of formalin produces an acute inflammatory nociceptive response [28,29]. In present study, the number of nociceptive responses were counted and totaled in 5 minute intervals for 60 minutes following formalin administration (5%, 50 μl). Saline-treated control rats displayed discrete biphasic behavioral responses consisting of an early short-lasting response (phase I, 0–10 minutes post-injection), followed by a late, prolonged response (phase II, approximately 16–60 minutes post-injection). These two phases were separated by a quiescent period (11–15 minutes post-injection) (Figures 1A and 1B) [28,29]. The duration of licking, lifting, and rubbing of the ipsilateral hind paw, which were considered to be nociceptive behaviors in the formalin model, peaked around 36–40 minutes after formalin intraplantar injection with maximal nociceptive behavior per minute of 32.6 ± 3.4 seconds, which was followed by a gradually decline (Figure 1A). The nociceptive behavior was compared between the saline- and EP [10, 50, and 100 mg/kg, intraperitoneal (i.p.)]-administrated rats. Nociceptive behavior by subcutaneous irritation during phase I was not different between the groups, but during phase II, was remarkably inhibited in the EP-administrated rats in a dose-dependent manner (Figure 1A). The total number of nociceptive responses during phase II after formalin injection in the saline pre-treatment group was 163.2 ± 18.6 seconds/minute. However, the total number of nociceptive responses was significantly decreased by pre-treatment of EP in a dose-dependent pattern (10 mg/kg, 117.0 ± 14.5; 50 mg/kg, 96.4 ± 11.2; 100 mg/kg, 74.0 ± 8.3; Figure 1B). I.p. injection of either saline or EP (100 mg/kg) alone, did not alter the behavior of the animals (data not shown). These results suggest that EP has an anti-nociceptive effect on formalin-induced inflammatory nociception.

### EP reduces formalin-induced paw edema

Intraplantar injection of formalin elicits significant inflammation (i.e. edema) in the center of the sole of the hind paw [30]. Therefore, to confirm whether the apparent attenuation of formalin-induced nociceptive behavior during phase II following EP administration reflected the effect of EP on the on-going peripheral inflammation produced by formalin injection, the size changes of the hind paw edema were compared between the saline- and EP-administrated rats (control, n = 15; 10 mg/kg, n = 14; 50 mg/kg, n = 10; 100 mg/kg, n = 13). To investigate the change of edema size, the foot thickness in the hind paw dorsal-plantar axis was determined by measurements with a fine caliper. As shown in Figure 1C, the thickness of the ipsilateral paw edema was increased 177.1 ± 6.3% compared to the thickness of the ipsilateral paw before formalin injection. However, the thickness of the paw edema in the formalin-evoked group was significantly reduced by administration of EP in a dose-dependent manner (151.4 ± 3.1% ~ 64.6 ± 3.3%) (Figure 1C).

### EP reduces formalin-induced c-fos expression in the spinal cord

**Figure 2:**
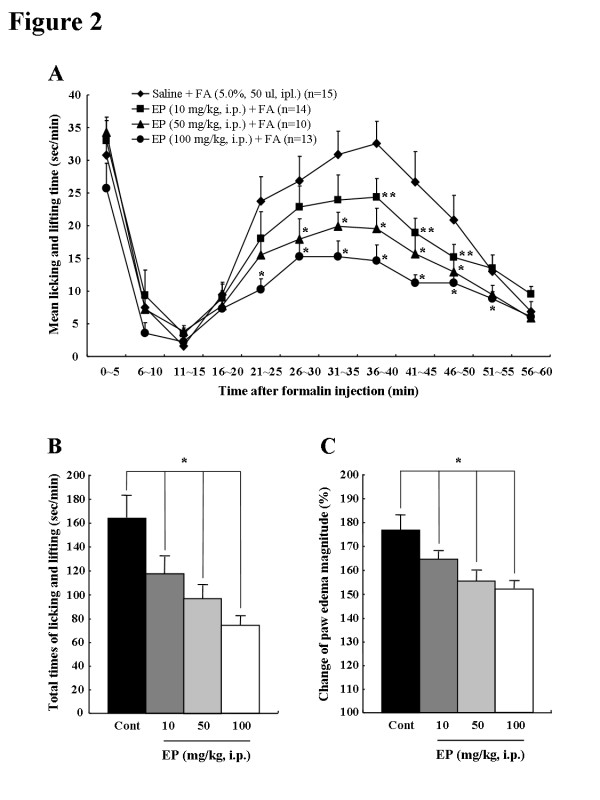
**Representative photomicrographs and graph showing immunoreactivity of c-Fos in the spinal DH (L4-5) following formalin injection. (A)** Western blots from spinal DH. N, normal. FA, saline-pretreated and formalin-treated rats, FA + EP, EP-pretreated and formalin-treated rats. E, EP alone. (B-D) Photomicrographs showing c-Fos expression in the spinal DH from normal rats **(B)**, saline-pretreated and formalin-treated rats **(C)**, EP-pretreated and formalin-treated rats **(D)**. The elevated number of c-Fos-immunorective (IR) cells produced by formalin was clearly decreased in both the superficial lamina (I-II) and deep lamina (III-IV) by EP-pretreatment. Insets are high magnification of the open rectangles. Scale bar = 100 μm. **(E) **The number of c-Fos-IR cells in spinal DH following EP pre-injection. The mean number of c-Fos-IR cells was calculated by averaging the total numbers per each region. Values are expressed as mean ± SEM. ^+^P < 0.01 vs. normal rats (saline-pretreated and saline-treated); *P < 0.01 vs. control rats (saline-pretreated and formalin-treated).

Because c-Fos, the protein product of the immediate-early gene c-fos, is a neuroactive marker that can be used to analyze nociceptive pathways [31-33], we compared the level of spinal c-Fos expression between the saline- or formalin-injected rats (n = 8/group) 36–40 minutes after formalin injection, the time at which the introduced formalin produced the maximal effects on nociceptive behavior (Figure 1A). c-Fos-immunoreactive (IR) cells were evaluated in the superficial laminae (I-II) and deep laminae (III-IV) of the DH in L4-L5 spinal cord where primary afferent fibers, from sciatic nerves innervating hind limb including the hind paw, form synapses with dorsal sensory neurons [34,35]. At 36–40 minutes after formalin injection, we confirmed the change of c-Fos expression in the ipsilateral DH of the L4-L5 spinal cord (Figure 2A). c-Fos expression was upregulated in the spinal DH of formalin-induced rats compare to normal, saline- pretreated and saline-treated rats. However, the elevated level of c-Fos expression was decreased by EP-administration (100 mg/kg, i.p.) (Figure 2A). In addition, we evaluated the anatomical distribution of c-Fos expression in spinal DH (Figures 2B2E). The c-Fos-IR in the L4-L5 spinal DH was very scarce in normal rats (I-IV, 28.9 ± 3.4; I-II, 11.3 ± 1.9; III-IV, 17.5 ± 1.8). The number of c-Fos-IR cells in the superficial and deep laminae was extensively increased following intraplantar injection of formalin (I-IV, 74.1 ± 3.4; I-II, 48.6 ± 2.3; III-IV, 25.6 ± 2.0), but the formalin-induced c-Fos-IR enhancement was significantly decreased by EP-administration (100 mg/kg, i.p.) 1 hour prior to formalin injection (I-IV, 45.9 ± 7.9; I-II,31.0 ± 6.4; III-IV, 14.9 ± 2.1) (Figures 2B2E). The number of c-Fos-IR cells in the contralateral DH was similar to that in the spinal DH of normal rats (data not shown). EP (100 mg/kg, i.p.), itself did not have any effect on c-Fos expression in the spinal cord. Taken together, the above results suggest that EP has an inhibitory action in spinal sensitization in formalin-induced acute inflammatory nociception (Figures 2B2E).

### EP attenuates formalin-induced neuronal p-ERK expression

**Figure 3:**
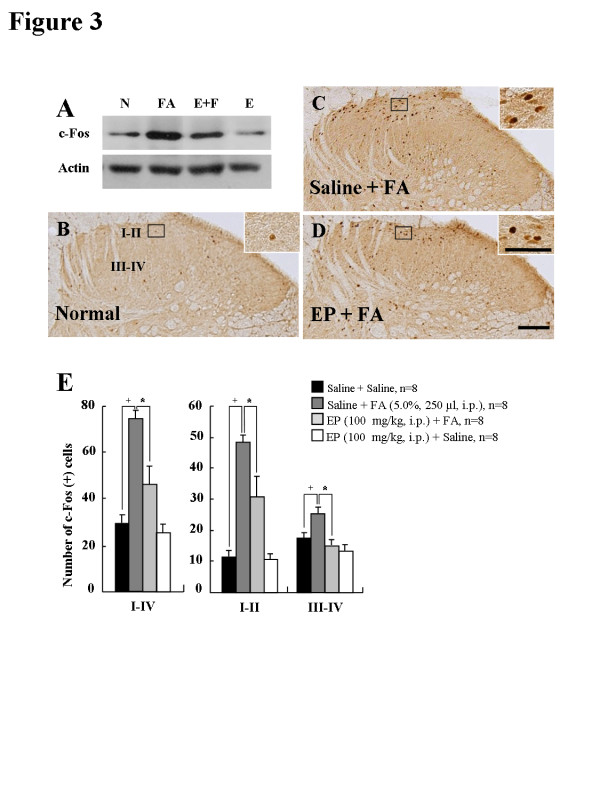
**Phosphorylation of ERK1/2 in the DH of spinal cord (L4-L5) after saline or EP pretreatment.(A)** Western blots from spinal DH. N, normal. FA, saline-pretreated and formalin- treated rats, FA + EP, EP-pretreated and formalin-treated rats. E, EP alone. (**B-D**) Photomicrographs showing c-Fos expression in the spinal DH from normal rats **(B)**, saline-pretreated and formalin-treated rats **(C)**, EP-pretreated and formalin-treated rats **(D)**. The number enhancement of p-ERK-IR cells produced by formalin was clearly decreased in both the superficial lamina (I-II) and deep lamina (III-IV) by EP-pretreatment. Insets are high magnification of the open rectangles. Scale bar = 100 μm. **(E)** The number of p-ERK-IR cells in spinal DH following EP preinjection. The enhancement in the number of p-ERK positive cells produced by formalin was significantly decreased in spinal DH by EP pre-administration. The mean number of p-ERK positive cells was calculated by averaging the total numbers per each region. Values are expressed as mean ± SEM. ^+^P < 0.01 vs. normal rats (saline-pretreated and saline-treated); *P < 0.01 vs. control rats (saline-pretreated and formalin-treated).

ERK 1/2 are expressed in the spinal cord and are activated in rat spinal DH neurons after inflammation [20,36]. Inhibitors of ERK signaling reduce nociceptive response in the phase II of the formalin test, suggesting a selective role for ERK 1/2 in nociceptive sensitization [20]. In addition, ERK phosphorylation is inhibited in the LPS-induced inflammation by EP administration [15]. Therefore, we investigated whether EP could produce its effects through the ERK 1/2 signaling pathway in the formalin-induced nociception. As illustrated in Figure 3A, at 36- 40 minutes after formalin treatment, we observed a clear phosphorylation of ERK 1/2 in the L4-L5 spinal DH. However, the elevated level of the phosphorylation of ERK 1/2 was decreased by EP administration (100 mg/kg, i.p.) (Figure 3A). Subsequently, we examined the spinal distribution of the phosphorylation of ERK 1/2 (Figures 3B3E). Immunohistochemical evaluation confirmed that p-ERK-IR cells in the L4-L5 spinal DH were very scarce in saline-administrated normal rats (I-IV, 12.6 ± 1.2; I-II, 12.3 ± 0.6; III-IV, 6.3 ± 0.3) (Figures 3B and 3E). The number of p-ERK-IR cells in lamina I-II of the spinal DH was significantly increased by formalin treatment (I-IV, 62.9 ± 7.2; I-II, 59.9 ± 2.7; III-IV, 19.2 ± 1.0), but these formalin-stimulated p-ERK enhancements were decreased by EP-administration (I-IV, 30.4 ± 3.8; I-II, 33.3 ± 2.2; III-IV, 13.7 ± 0.6) (Figures 3C3E).

**Figure 4:**
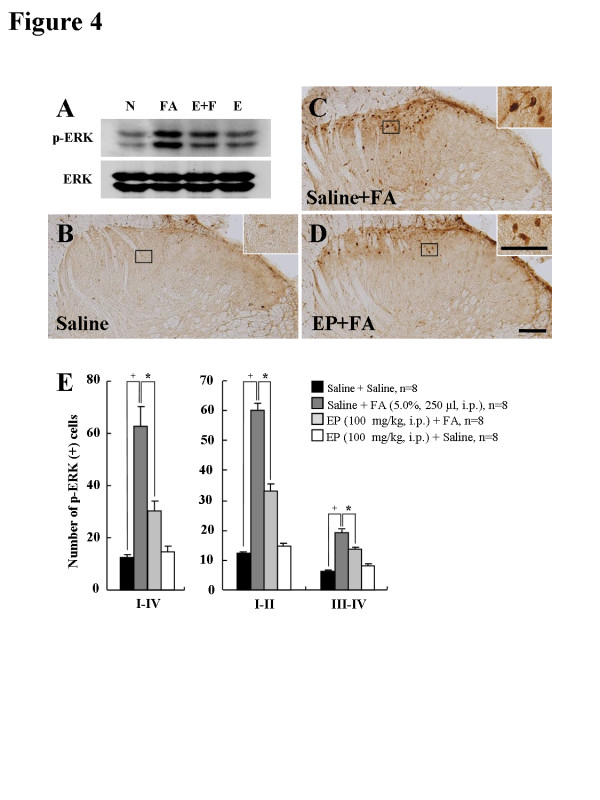
**Representative photomicrographs showing p-ERK-IR in the spinal DH (L4-L5) of formalin-treated rats. (A-I)** p-ERK-IR in the spinal DH of the saline-pretreated and formalin treated rats. The p-ERK-IR was found exclusively in spinal DH neurons** (A-C)**, but not in microglia **(D-F)** or astrocytes **(G-I)**. **(J,K)** Microglia** (J) **and astrocytes **(K)** in normal rat (salinepretreated and saline-treated). Scale bar = 50 μm.

To investigate the nature of the p-ERK-IR cells, we examined whether the ERK 1/2 are activated in neurons, microglia, or astrocytes using a multiple immunofluorescence method. Interestingly, the p-ERK immunofluorescence in the spinal DH was found exclusively in neurons (83.1%; 103 p-ERK-IR and NeuN-IR neurons of 124 p-ERK-IR neurons) (Figures 4A-4C), but not clear in microglia or astrocytess (Figures 4D-4I). Also microglia and astrocytes were not sufficiently activated 36–40 minutes after formalin treatment (Figures 4E, 4H, 4J and 4K). These results suggest that EP attenuates the formalin-induced acute inflammatory nociception through the inhibition of neuronal ERK activation, but not glial ERK activation.

Because p38 and c-Jun, N-terminal kinase (JNK) MAPKs are activated in microglia and astrocytess, respectively, after a variety of nerve damage, and both MAPKs also contributes to the development and maintenance of various forms of nociception [21,27,37], we also investigated whether both MAPKs are regulated by formalin or EP. Unlike the very low level of basal p-ERK, moderate basal p-p38 and p-JNK were evident in the spinal DH. Nonetheless, p- p38 and p-JNK were not increased or inhibited by hind paw formalin injection or EP administration into the peritoneal cavity ( Additional file1: Figure S1).

### Microglia are not morphologically activated or inhibited by formalin or EP

Spinal microglia are activated in inflammatory and neuropathic pain [38], and EP attenuates inflammation through the inhibition of microglial activation in various neurological disease models [18,28]. Therefore, we examined whether spinal microglia are activated 36–40 minutes after formalin injection (i.e., when formalin produces the maximum nociceptive effects on formalin-induced pain) and if so, whether the activated spinal microglia is inhibited by EP administration. Activated microglia usually display CD11/b- or Iba-1 (a marker for microglia/macrophage lineage cells)-IR with enlarged cell bodies and much shorter and thicker processes [28,39]. However, when we analyzed CD11/b-IR cells 36–40 minutes following formalin injection, microglial activation by formalin and EP-induced inhibition were not clearly evident in ipsilateral DH compared to normal spinal DH (Figures 4E and 4J). The results were consistent with previous studies [28,30,40], which reported that at least 1 day was required for the expression of OX-42-IR.

**Figure 5:**
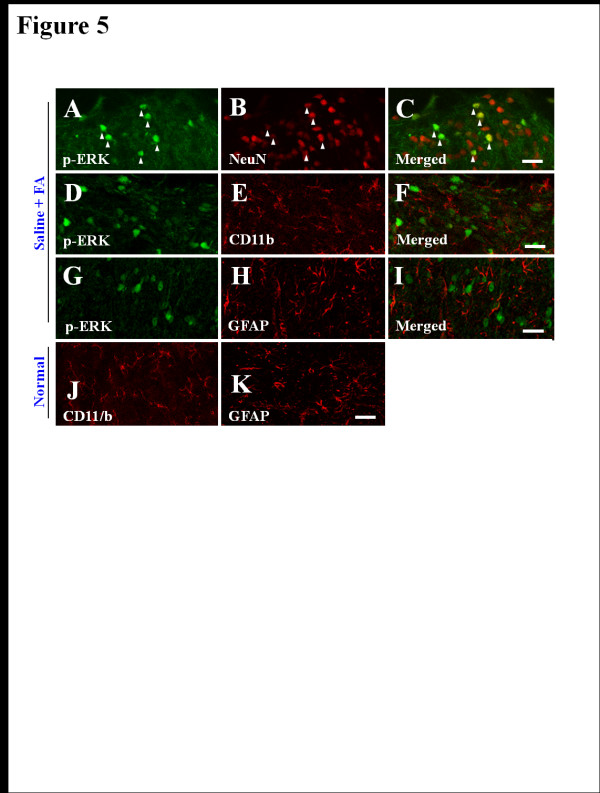
**Photomicrographs showing changes in CD11/b-IR in the spinal DH (L4-L5) 3 days after formalin injection.** Inhibitory effects of EP on microglial activation were clear at 3 days following formalin injection. Insets are high magnification of the open rectangles. **(A)** Contralateral spinal DH of saline-pretreated and formalin-treated rats. **(B)** Ipsilateral spinal DH of saline-pretreated and formalin-treated rats.** (C) **Ipsilateral spinal DH of EP-pretreated and formalin-treated rats. Scale bar = 50 μm. **(D)** The Iba-1-IR intensity was measured as the average pixel intensity per 0.5 mm^2^ area within medial portion of the L4-L5 spinal DH at 3 days after formalin injection. The Iba-1-IR intensity was significantly increased by formalin injection, however this increased intensity significantly decreased by EP. Values are expressed as mean ± SEM. *P < 0.01 vs. normal rats (saline-pretreated and saline-treated) or control rats (saline-pretreated and formalin-treated).

To examine whether the EP could inhibit microglial activation in our formalin-induced inflammatory nociception model, we administrated EP (100 mg/kg, i.p.) to formalin-injected rats once daily for 3 days. When we analyzed Iba-1-IR in spinal DH 3 days following formalin injection, microglia was clearly activated by formalin intraplantar injection compared to that of saline-treated rats. However, this microglial activation was remarkably inhibited by EP administration (Figures 5A-5D). These results confirmed that spinal microglia was not affected in cell morphology by either formalin or EP during phase II of the formalin-induced pain model, and that spinal microglia do not contribute to acute inflammatory pain.

### I.T. Administration of PD-98059 reduces formalin-induced inflammatory nociception

**Figure 6:**
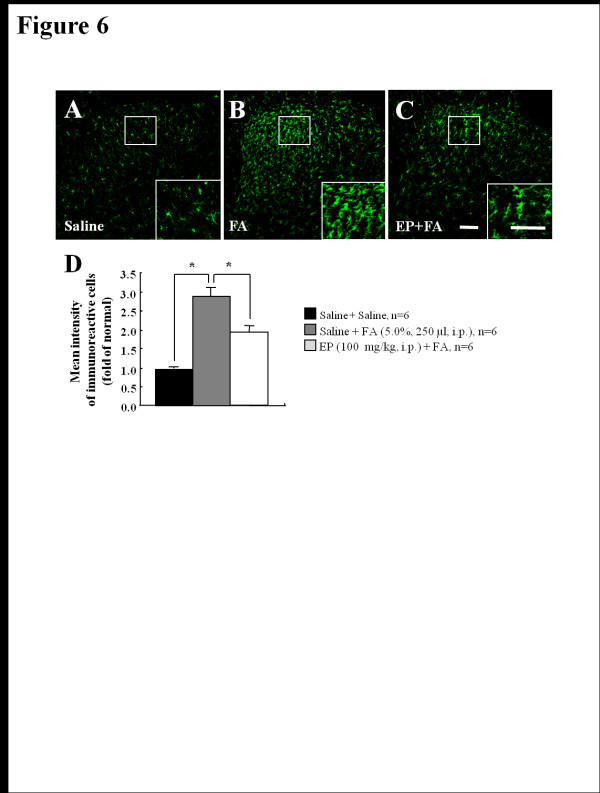
**Intrathecal administration of PD-98059 inhibits formalin (5%, 50 μl, i.p.)-induced inflammatory nociception. (A)** Time course of formalin-induced nociceptive behavior. The nociceptive behavior by formalin injection was significantly inhibited by intrathecal administration of PD-98059 in a dose dependent manner. Values are expressed as mean ± SEM. *P < 0.01, and **P < 0.01 vs. control rats (vehicle-pretreated and formalin-treated). **(B)** Total times of nociceptive behavior were remarkably blocked during phase II, but not during phase I by intrathecal administration of PD-98059 in a dose-related fashion following intraplantar injection of formalin. Values are expressed mean ± SEM. *P < 0.01 vs. control rats (vehicle-pretreated and formalin-treated).

After intraplantar injection of formalin, nociceptive behavior increased and p-ERK expression was up-regulated, mainly in DH neurons of L4-L5 spinal segments, but not in microglia and astrocytes. The elevated nociceptive response and p-ERK expression were remarkably reduced by i.p. administration of EP (Figures 1 and 3). These results support the hypothesis that neuronal p-ERK expression may contribute to formalin-induced nociception. To address this issue, we directly introduced the MEK inhibitor, PD-98059, to subarachnoid space of normal rats. In the vehicle-treated rats, the duration of nociceptive response by formalin stimulation peaked at 36–40 minutes (34.4 ± 5.3 seconds/minute), and then gradually declined. Total duration of nociceptive behavior during phase II was 213.0 ± 32.7 seconds (Figures 1A and 1B) similar to the result of Figure 1B. However, these nociceptive responses were almost completely blocked by the i.t. administration of PD-98059 in a dose-dependent manner in peak time (5 μg, 19.7 ± 5.8 seconds/minute; 10.0 μg, 11.3 ± 5.0 seconds/minute), and total duration of nociceptive behavior (5 μg, 96.0 ± 26.7 seconds/minute; 10.0 μg, 64.0 ± 19.1 seconds/minute) during phase II was also decreased (Figures 6A and B). These results indicate that i.t. introduction of PD-98059 inhibits formalin-induced inflammatory pain.

## Discussion

In the current study, we investigated the possibility that EP may be potential analgesic for formalin-induced inflammatory nociception. When EP was administrated intraperitoneally 1 hour before formalin injection into the plantar surface of the hind paw, it attenuated nociceptive behavior, the size of hind paw edema (Figure 1), and the activation of c-Fos and ERK in the neurons of L4-L5 spinal DH (Figures 2,3,4), which is considered a consequence of its central and peripheral pharmacological actions. In addition, the i.t. introduction of the MEK inhibitor, PD-98059, reduced formalin-induced inflammatory nociception (Figure 5). These data indicate that neuronal ERK phosphorylation is involved in the acute inflammatory nociceptive mechanism, and the EP can attenuate acute inflammatory nociception by inhibiting neuronal ERK activation in spinal DH.

Subcutaneous hind paw injection of formalin elicits two-phase nociceptive responses. While phase I is considered to reflect acute nociceptive pain by a direct stimulation of the nerve by the formalin, phase II is attributed to the combination of ongoing inflammatory-related afferent input from peripheral tissue and functional changes in the spinal DH (central sensitization) [29,41]. In the current study, administration of EP clearly reduced the size of hind paw edema by formalin stimulation and nociceptive behavior during phase II, but not during phase I (Figure 1A). And it has been demonstrated that most peripheral inflammation is often accompanied by a variety of pain [42], and that EP seems to exert pharmacological effects, such as suppression of inflammation (i.e., severe sepsis, acute pancreatitis) [2]. It also has been reported that EP has an anti-inflammatory effect in the nervous system by inhibiting microglial activation in models of stroke and neural damage [6,7,17,18,43]. Based on these collective findings, we suggest that EP could produce anti-nociceptive effect by regulating peripheral and/or central mechanisms underlying formalin-induced inflammatory nociception.

Intraplantar injection of formalin produces a massive inflammatory response at the injection site [29], thereby causing paw edema [30]. To verify the peripheral effect of EP, we examined the changes of hind paw edema 1 hour following formalin injection. When rats were given EP injection 1 hour before formalin injection, the thickness of hind paw edema was significantly decreased (12.5 ~ 25.7%) compared to that of animals treated with formalin alone (Figure 5). Reduction of the formalin-induced paw edema by EP (Figure 1C) suggests its clear anti- edematous effects in the inflammatory site. Several mechanisms could explain the anti-edematous effects of EP for formalin-induced inflammation. First, EP may inhibit the activation and recruitment of peripheral immune cells to formalin-induced inflammatory site. Jang et al. [44] recently demonstrated that EP has the ability to inhibit neutrophil activation, inflammatory cytokine (TNF-α, IL-1β) release, and nuclear factor κB (NF-kB) translocation in ischemia/reperfusion-induced heart injury. Second, EP may inhibit peripheral inflammation such as adenosine. It has been demonstrated that the i.t. administration of the adenosine receptor agonist, cyclohexyladenosine (5 μg/kg), suppresses peripheral inflammation by decreasing neutrophil infiltration into skin lesions [45]. Third, like botulinum toxin A, EP may reduce neurogenic inflammation in the inflamed skin by reducing the releasing of neurotransmitters such SP, CGRP and glutamate from peripheral sensory nerve terminals by formalin injection. Released neurotransmitters contribute to the formalin-induced edema [46]. Peptide-mediated transdermal delivery of botulinum neurotoxin type. A reduces neurogenic inflammation in the skin [47]. The detailed cellular and molecular mechanisms underlying the anti-edematous effects of EP in the periphery remain to be elucidated. The detailed cellular and molecular mechanisms underlying the anti-edematous effects of EP in the periphery remain to be elucidated.

To verify the possible central mechanism of EP, we examined the changes in c-Fos expression in the spinal DH during phase II (the peak time point of nociception; 36–40 minutes after formalin injection) of formalin-induced nociception. In agreement with our previous report [28], the increase in formalin-induced c-Fos expression was mainly observed in the L4-L5 superficial and deep laminae where the primary nociceptive afferents from spinal nerve terminate (Figure 2). However, the upregulation of c-Fos expression by formalin stimulation was clearly inhibited by EP (Figure 2). Because c-Fos is expressed in the spinal cord subjected to many kinds of peripheral noxious stimulation [31-33], the reduction of c-Fos expression in the spinal DH clearly indicates an anti-nociceptive role of EP.

Accumulating evidence shows that MAPKs (ERK, p38, and JNK) pathways contribute to pain sensitization after tissue/nerve injury via distinct molecular/cellular mechanisms [24-27]. In particular, ERK mediates intracellular signal transduction in response to a variety of stimuli. The phosphorylation of ERK in the nociceptive neurons of spinal DH occurs in response to axotomy, electrical stimulation to the peripheral nerve, noxious stimulation of the peripheral tissue, and peripheral inflammation [20,27]. The phosphorylation of ERK plays a critical role in central sensitization by regulating the activity of glutamate receptors and potassium channels, and inducing gene transcription, and thereby contributes to persistent inflammatory and neuropathic pain [27]. These reports suggest that the materials regulating the phosphorylation of ERK could control nociceptive mechanism. Presently, ERK was phosphorylated in mainly neurons of L4-L5 spinal DH by formalin-injection; 83.1% of p-ERK IR cells were NeuN-IR cells (Figure 4). However, the elevated p-ERK expression by formalin injection was clearly attenuated by EP administration (Figure 3). In accordance with these results, it was recently reported that EP can suppress the phosphorylation of ERK in LPS-stimulated BV2 cells [15]. These results indicate that EP could inhibit inflammatory nociception by regulating the phosphorylation of ERK in neurons of spinal DH after formalin injection.

I.t. injection of the MEK inhibitor, PD-98059, blocks the central sensitization-mediated phase II of the painful response to formalin injection [27,48]. MEK dominant negative mutant mice in which MEK function is suppressed exclusively in neurons show decreased phase II responses in the formalin-induced nociception [49]. The MEK inhibitor, U0126, also blocks secondary mechanical hypersensitivity from central sensitization following intraplantar injection of capsaicin [50]. In addition, i.t. injection of MEK inhibitors inhibits inflammatory thermal/mechanical hypersensitivity following intraplantar injection of bee venom [51] and CFA [19,52], and in a model of monoarthritis and inflammatory visceral pain [26,53]. I.t. MEK inhibitors can also suppress neuropathic pain by streptozotocin-induced diabetes and spinal cord injury [54,55]. Presently, we verified that PD-98059 completely blocked formalin-induced nociception during phase II (Figure 6). These results suggest that the phosphorylation of spinal ERK could play a critical role in development and maintenance of formalin-induced inflammatory nociception.

Because EP has an anti-inflammatory effect in the nervous system by inhibiting the microglial activation in a model of stroke and excitotoxic neuronal damage, and in a LPS-stimulated in vitro model [6,7,17,18,43], we can speculate that central pharmacological roles of EP could target the microglial activation of spinal DH following formalin injection. However, morphological changes of CD11/b-IR microglia was not clearly observed at the peak time of nociception, 36–40 minutes after formalin injection, and the CD11/b-IR microglia was also not affected by EP at the same time, indicating that EP produced its maximal effect. Microglia was sufficiently activated 3 days after formalin injection, and the activated microglia was completely inhibited by EP (Figure 5). These findings were consistent with previous reports that CD11/b- or OX-42-IR microglia are not distinctly activated as early as 1 hour following formalin injection, but only increase after 1 day and peak 7 days following formalin injection [28,30,40,56]. Thus, the collective findings suggest that microglial activation might not directly contribute to the anti-nociceptive effects of EP on the early stage of formalin-induced nociception.

After nerve damage, the three MAPKs are differentially activated in spinal neurons and glial cells by various postsynaptic receptors and multiple protein kinases, and the activated glial cells induces the synthesis of pronociceptive and proinflammatory mediators that act to develop and maintain pain [27]. ERK integrates multiple signaling pathways and regulates the Kv4.2 potassium channel in the spinal cord, and contributes to the induction and maintenance of central sensitization via posttranslational and transcriptional regulation, respectively [27]. ERK is activated in neurons from 10 minutes to 6 hours, in microglia on day 2, in both microglia and astrocytes on day 10, and in astrocytes on day 21 after spinal nerve ligation [24]. In agreement with these reports, we confirmed that ERK was phosphorylated mainly in the spinal neurons, but not microglia and astrocytess at 36–40 minutes after formalin injection, and that the elevated ERK phosphorylation was inhibited by EP. These results suggest that the inhibition of neuronal phosphorylation of ERK in spinal DH might be associated with anti-nociceptive effects produced by EP. Compared to mechanisms of ERK pathway in neuropathic pain, neuronal and glial mechanisms of ERK for inflammatory nociception control remain to be elucidated. p38 and JNK are phosphorylated in primarily spinal microglia and astrocytes, respectively, after peripheral inflammation and peripheral nerve injury [57-59]. The activation of the p38 and JNK in microglia and astrocytes is critical for the maintenance of inflammatory/neuropathic pain. Therefore, we investigated whether the phosphorylation of p38 and JNK were increased in spinal DH at peak time point of nociceptive behavior after formalin injection. Expression of p-p38 and p-JNK was not increased or decreased in spinal DH by formalin injection or EP administration ( Additional file 1: Figure S1). These findings are supported by that p-p38 begins to increase at 12 hours, reaches a peak at 3 days after a spinal nerve ligation, and is maintained at elevated levels even after 3 weeks [24,57], and that p-JNK is persistently increased in spinal astrocytes at 1, 3, 10, and 21 days after spinal nerve ligation [59] and partial sciatic nerve injury [21]. Therefore, our findings suggest that p38 and JNK might not directly contribute to the development and maintenance of hypersensitivity in the formalin-induced nociception. Interestingly, recent publications reported contradictory results in the expression time of p-p38; p38 was rapidly activated in the spinal microglia minutes following intrathecal administration of substance P or intradermal injection of formalin and the activation persisted for 1 hour [38,60]. In addition, induction of a secondary increase of p-p38 expression in spinal microglia occurred and was maximal 3 to 7 days after injection [60]. The exact role of p-p38 and microglia in inflammatory pain are still unclear. Future studies should investigate the clear function of activation of MAPKs signaling pathway in various pains.

## Conclusions

While increased spinal ERK phosphorylation is important for pain behaviors based on the MEK inhibitor studies, the direct link between EP’s inhibitory effects in inflammatory nociceptive responses and its modulating effects on p-ERK expression has not been established. In this study, EP attenuated the inflammatory nociceptive response, the size of hind paw edema and the activation of spinal c-Fos and ERK 1/2 in the formalin-induced inflammatory pain. And, the i.t. introduction of MEK inhibitor PD-98059 reduced the nociceptive behavior by formalin. These results strongly suggest that EP has an anti-nociceptive effect on formalin-induced inflammatory pain by inhibiting the neuronal ERK activation in spinal DH.

## Materials and methods

### Animals

All experiments were approved by the Institutional Animal Care and Use Committee (IACUC) in College of Oriental Medicine, Kyung Hee University. And animal treatments were performed according to the ethical guidelines of the International Association for the Study of Pain for the investigation of experimental pain in conscious animals [61]. The male Sprague–Dawley (SD) rats (weight, 250–280 g) were kept at a constant temperature of 23 ± 3°C with a 12-h light–dark cycle (light on 08:00 to 20:00), and fed food and water ad libitum. The animals were allowed to habituate to the housing facilities for 1 week before the experiments.

### Formalin-induced behavioral test

**Figure 7:**
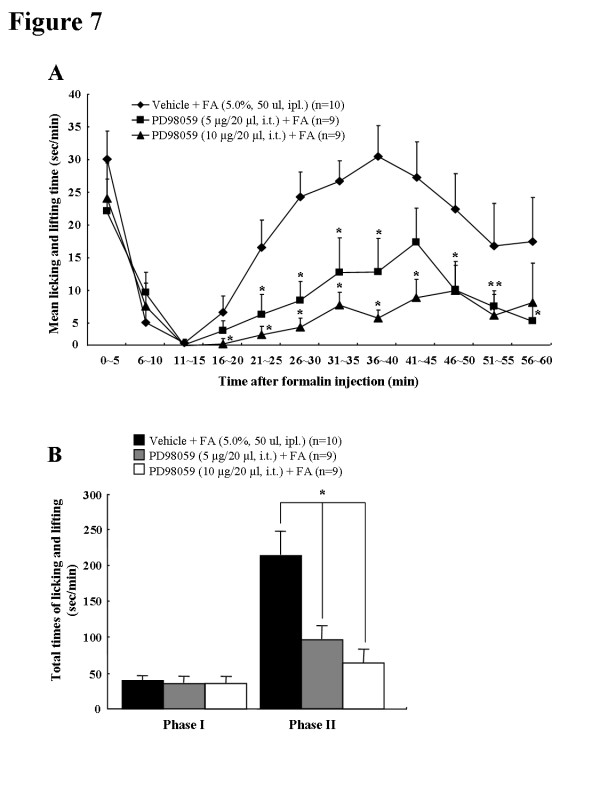
**Schematic drawing representing the experimental protocol used for inflammatory pain with formalin, ethyl pyruvate (EP) and PD-98059 treatments.** IHC, immunohistochemistry; WB, Western blots; i.pl., intraplanter; i.p., intraperitoneal.

The formalin-induced nociceptive response was tested as described previously [28]. Briefly male SD rats were randomly assigned to two groups; saline-treated control group (n = 15), and EP-treated experimental group (n = 37). Three EP-treated group received EP (Sigma-Aldrich, USA) at doses of 10 mg/kg (n = 14), 50 mg/kg (n = 10) or 100 mg/kg (n = 13) intraperitoneally 1 hour before formalin injection, respectively (Figure 7A). Control rats received an equal volume of saline vehicle. The dosage of EP was determined based on doses used in previous reports of the therapeutic effects of EP [9,12,62]. The time point of formalin injection was determined based on a previous report of the optimal delivery of EP in rats [9,12,62]. Following intraplantar injection of formalin (5%, 50 μl) into the right hind paw, rats were placed in a clear plastic cage (20 × 26 × 12 cm) without bedding, and the total time of pain responses, licking/rubbing on the injected area or lifting the paw, was counted in 5 minutes interval for 60 minutes. The behavioral tests were performed blinded under the constant condition (temperature, 23 ± 3°C; humidity, 55 ± 5%) between 9:00 am and 12:00 am in a quiet room.

### Measurement of paw edema

For the measurement of paw edema, we adopted the method described previously [28,30,63]. The foot thickness in the dorsal-plantar axis was measured with a fine caliper (Mitutoyo, Japan) before and 1 hour after formalin injection (Figure 7A). And the index of paw edema was calculated as the mean difference of paw thickness (thickness of the ipsilateral paw after formalin injection/thickness of the ipsilateral paw before formalin injection × 100). Evaluation of paw edema was also performed by an experimenter unaware of the experimental condition.

### Immunocytochemical evaluation

The anti-nociceptive effect of EP (100 mg/kg, i.p.) peaked around 36–40 minutes after intraplantar injection of formalin. So, at that time following formalin injection, rats for immunohistochemical evaluation (n = 8 per group) were anesthetized i.p. with 40 mg/kg sodium pentobarbital, and perfused with fresh 4% paraformaldehyde in 0.1 M phosphate buffer (pH 7.4) (Figure 7A). The L4-L5 spinal segments were removed and postfixed at 4°C overnight and then cryoprotected in 0.1M PBS (pH 7.4) containing 30% sucrose for 48 hours at 4°C. Immunostaining was carried out according to previously established procedures [28,39,64]. Briefly, eight transverse sections (30 μm thickness) in 500 μm interval selected from each animal were incubated for 30 minutes with 3% H_2_O_2_ in 0.1M PBS (pH 7.4) to remove endogenous peroxidase activity, and then blocked with solution containing 5% normal goat/or horse serum, 2% BSA, 2% FBS and 0.1% triton X-100 for 2 hours at room temperature (RT). The sections were incubated overnight at 4°C with either rabbit anti-c-Fos (1:10,000; Oncogene, U.S.A.), or rabbit anti-phospho (p)-ERK (1:500; Cell Signaling, U.S.A.), and then washed in PBS. Sections were then incubated with biotinylated secondary antibodies (Vector Laboratories, Burlingame, CA) at a dilution of 1:200 for 1 hour at RT, followed by incubation with avidin and biotinylated HRP complex (Vector Laboratories) at 1:200 for 1 hour at RT. All sections were visualized with 3,3′-diaminobenzidine (D-5637; Sigma, U.S.A.). The immunostained sections were mounted onto gelatinized glass slides, dehydrated through a series of ethanol, cleared, and cover-slipped with permount. Images of stained sections were visualized and captured using a digital microscope system (DP70, Olympus, Japan) under light microscope. Superficial laminae (I-II) and deep laminae (III-IV) were outlined, and the c-Fos- or p-ERK-immunoreactive (IR) cells were counted. Evaluation of the immunostained sections was performed by an experimenter unaware of the experimental condition.

### Immunofluorescence evaluation

For double immunofluorescent staining, sections were incubated overnight at 4°C with a mixture of rabbit anti-p-ERK antibody and mouse anti-NeuN (1:500; Chemicon, U.S.A), or rat anti-CD11/b, or mouse anti-GFAP (1:1,000; Chemicon, U.S.A) antibody. The sections were then incubated for 1 hour at RT with mixture of Cy3- and FITC-conjugated rabbit/rat/mouse IgG antibody (1:200; Jackson ImmunoResearch, U.S.A.), and then examined with confocal imaging system (LSM 5 PASCAL; Carl Zeiss, Germany). Immunofluorescence images for Iba-1 antibody were analyzed as described previously [65]. In brief, the images were captured using confocal microscopy and changes in immunofluorescence intensity of Iba-1 expression in the spinal DH after formalin injection were quantified by measuring the average pixel intensity per 0.5 mm^2^ area within the medial portion of the superficial and deep laminae of the spinal DH in four sections per rat at the level of L4-5 spinal segments 3 days after formalin injection.

### Western blot analysis

To investigate the level of protein expression, EP (100 mg/kg) was injected i.p. 1 hour before the intraplantar injection of formalin. And at 36–40 minutes after formalin injection, L4-L5 spinal segments were removed with lysis buffer (50 mM Tris-Cl, pH 7.5, 150 mM NaCl, 1% Triton X-100, 10% glycerol, and protease inhibitor mixture) (Figure 7A). A total of 50 μg of tissue lysate from each sample was resolved by electrophoresis on a 10% SDS-PAGE. The proteins were then transferred to PVDF membranes and blocked with 5% nonfat dry milk in Tween 20-containing Tris-buffered saline (TBST, 20 mM Tris, pH 7.4, 0.1% Tween 20, and 150 mM NaCl). The membranes were probed overnight with primary antibody (p-ERK, p-p38, or p-JNK at 1:2,000; Cell Signaling, U.S.A.) at 4°C, which was followed by incubation with HRP-conjugated secondary antibody at RT for 1 hour prior to ECL treatment and exposure to X-ray film. For normalization of antibody signal, the membranes were stripped and reprobed with antibodies for ERK 1/2 or actin.

### Intrathecal (I.T.) administration of PD-98059

The i.t. injections were performed under light isofluran anesthesia (1-2%). The dorsal fur of each rat (saline + formalin, n = 10; 5 μg of PD-98059 + formalin, n = 9; 10 μg of PD-98059 + formalin, n = 9) was shaved, the spinal column was arched, and a 30-gauge needle was directly inserted into the subarachnoid space, between the L5 and L6 vertebrae [66]. Correct i.t. positioning of the needle tip was confirmed by manifestation of a characteristic tail flick response. The 5 and 10 μg of the ERK upstream kinase (MEK) inhibitor PD-98059 (2-amino-3′-methoxyflavone; Calbiochem, USA), or vehicle (saline alone or 10% dimethylsulfoxide) were slowly injected into the rat with a 50 μl Hamilton micro syringe in a total volume of 5 μl. The entire injection procedure, from the induction of anesthesia until recovery of consciousness, took 4–5 minutes. Preliminary injections were performed with a similar volume of 10% India ink solution and the reliability and accuracy of this method was confirmed by subsequent dissection of the lumbar spinal cord. The success rate for the prior injections with this technique was over 97.5%. The same investigator performed all injections. The intraplantar injection of formalin and behavioral test were performed 20 minutes after i.t. injection of PD-98059 as described above (Figure 7B).

### Statistical analysis

The statistical significance of differences between the values was determined using the ANOVA with a Fisher's post hoc test. All data are presented as the mean ± S.E.M. and a statistical difference was accepted at the 5% level unless indicated otherwise.

## Competing interest

All the authors of this manuscript have no conflict of interest in this subject.

## Authors’ contributions

MJL performed the behavioral experiment, immunohistochemistry and Western blot, and prepared figures. MJ assisted with behavioral experiments. SHK and HSJ participated in the design of the study. IHC conceived all experiments, analyzed the results, and wrote the manuscript. All authors have read and approved the final manuscript.

## Supplementary Material

Additional file 1 **Figure S1.** Phosphorylation of p-p38 and p-JNK in the DH of spinal cord (L4-L5) after saline or EP pretreatment. N, normal rats (saline pretreated + saline treated); FA, saline pretreated + formalin treated; E + F, EP (100 mg/kg, i.p.) pretreated + formalin treated; E, EP (100 mg/kg, i.p.) treated. Click here for file
