# Supplementary figures and images for: Ethyl pyruvate attenuates formalin-induced inflammatory nociception by inhibiting neuronal ERK phosphorylation
Source: Mol Pain. 2012 May 28;8:40. doi: 10.1186/1744-8069-8-40 (PMC3472237; doi:10.1186/1744-8069-8-40)

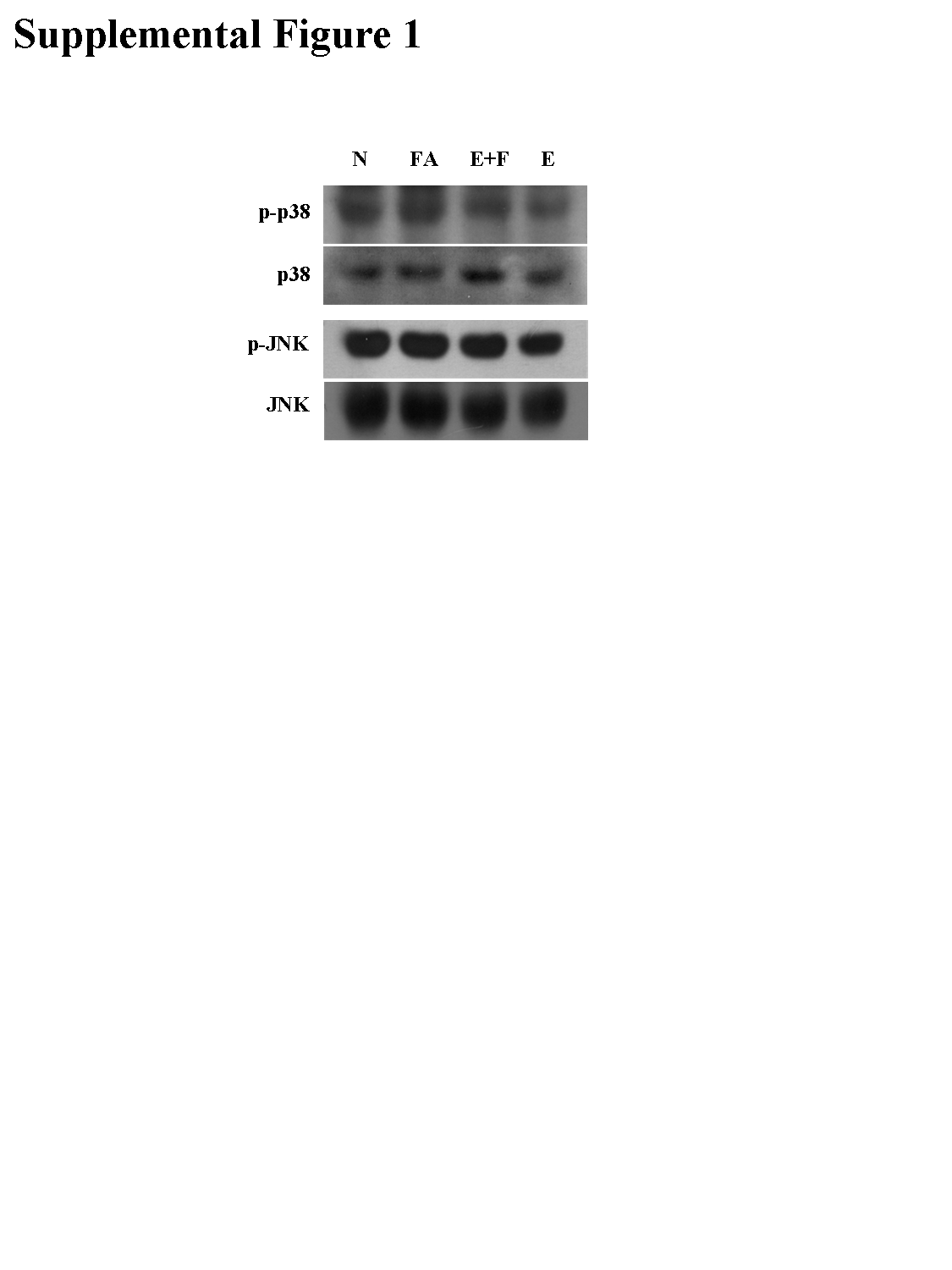

Supplement: Additional file 1 — Figure S1. Phosphorylation of p-p38 and p-JNK in the DH of spinal cord (L4-L5) after saline or EP pretreatment. N, normal rats (saline pretreated + saline treated); FA, saline pretreated + formalin treated; E + F, EP (100 mg/kg, i.p.) pretreated + formalin treated; E, EP (100 mg/kg, i.p.) treated. [file 1744-8069-8-40-S1.tiff]
